# Non-invasive physical plasma activates stimulator of interferon genes pathway in triple negative breast cancer and is associated with increased host immune response

**DOI:** 10.3389/fimmu.2025.1631530

**Published:** 2025-07-24

**Authors:** Guilin Wang, Marcel Arnholdt, André Koch, Markus D. Enderle, Markus Hahn, Sara Y. Brucker, Martin Weiss

**Affiliations:** ^1^ Department of Women’s Health, University of Tübingen, Tübingen, Germany; ^2^ Department of General Surgery (Breast Surgery), the Affiliated Hospital of Southwest Medical University, Luzhou, China; ^3^ Erbe Elektromedizin GmbH, Tübingen, Germany; ^4^ NMI Natural and Medical Science Institute, Reutlingen, Germany

**Keywords:** non-invasive physical plasma (NIPP), cold atmospheric plasma (CAP), argon plasma devitalization (APD), plasma-treated solution (PTS), triple negative breast cancer

## Abstract

Triple-negative breast cancer (TNBC), characterized by the absence of ER, PR, and HER2 receptors, remains one of the most aggressive breast cancer subtypes, with limited therapeutic options and a high relapse rate. While immune checkpoint inhibitors (ICIs) have shown promise by leveraging TNBC’s immunogenic profile, their use is often accompanied by significant toxicity, necessitating the development of safer immunomodulatory strategies. Non-invasive physical plasma (NIPP), a novel low thermal plasma technology that can be generated using various gases, including argon, and producing reactive oxygen and nitrogen species (RONS), has emerged as a potential alternative. This study investigates the capacity of direct (argon plasma devitalization, APD) and indirect (plasma-treated solution, PTS) plasma modalities to induce cytotoxicity and activate immune signaling via the stimulator of interferon genes (STING) pathway in TNBC. Dose-dependent RONS generation by APD and PTS correlated with reduced viability and apoptosis induction in MDA-MB-231 TNBC cells. Both plasma modalities caused DNA damage and upregulated key proteins in the STING pathway, including γ-H2AX, p-STING, and p-TBK1, with sustained activation observed up to 24 hours post-treatment. Furthermore, STING-dependent transcription of IFN-β and interferon-stimulated genes (ISGs) confirmed the immunogenic potential of NIPP. Conditioned media from plasma-treated TNBC cells induced M1 polarization in THP-1-derived macrophages, an effect significantly reduced upon specific STING inhibition with H-151. The immunomodulatory effects of NIPP were validated in patient-derived TNBC organoids, where plasma treatment disrupted organoid structure, reduced viability, and promoted M1 macrophage polarization. Collectively, these findings highlight the dual cytotoxic and immunostimulatory potential of NIPP in TNBC through STING pathway activation, claiming it as a promising, low-toxicity component in combination with conventional immunotherapy.

## 1 Introduction

Triple-negative breast cancer (TNBC) is a breast cancer subtype characterized by the absence of estrogen receptor (ER), progesterone receptor (PR), and human epidermal growth factor receptor 2 (HER2) expression (1). Due to the lack of these molecular markers and its high heterogeneity, TNBC lacks established targets for precision therapies, resulting in the poorest prognosis among breast cancer subtypes. Chemotherapy remains the cornerstone of systemic treatment for TNBC. However, approximately half of TNBC patients experience relapse despite initial successful treatment, highlighting the urgent need for alternative therapeutic approaches (2).

Modulating the tumor immune microenvironment (TIME) has emerged as a promising strategy in cancer therapy. TNBC is characterized by an increased infiltration of immune cells, including tumor-infiltrating lymphocytes (TILs), macrophages, and cancer-associated fibroblasts, rendering it more immunogenic compared to other breast cancer subtypes (3, 4). This immunogenicity makes TNBC a suitable candidate for immunotherapy. The clinical success of immune checkpoint inhibitors (ICIs), such as pembrolizumab, has demonstrated the feasibility of this approach in TNBC treatment (5, 6). However, the addition of checkpoint inhibitors to standard chemotherapy has increased the incidence of severe adverse events by more than sixfold (6). Therefore, there is an urgent need to develop alternative strategies that effectively activate the immune system while minimizing severe toxicity.

Non-invasive physical plasma (NIPP) is an emerging medical technology that generates a highly energized gas at room temperature and atmospheric pressure (7). This ionized gas comprises reactive oxygen and nitrogen species (RONS), charged ions, low-dose UV radiation, visible light, and electrons, exerting diverse biological effects (8). NIPP can be administered either directly or indirectly. Direct application involves direct plasma exposure to the target (APD), whereas indirect application utilizes plasma-treated solutions (PTS), in which a medium is first treated by plasma and then subsequently applied to the target (9–11). PTS enables plasma-based treatment in areas where direct exposure is not feasible. However, short-lived reactive species, electromagnetic fields, cannot be preserved in PTS, potentially leading to differences in the biological effects of direct and indirect plasma treatments. Recently, preclinical studies have demonstrated that NIPP exhibits anti-cancer effects and indicate its potential to stimulate anti-tumor immune responses (12–14).

The stimulator of interferon genes (STING) pathway plays a crucial role in orchestrating immune responses in the presence of aberrant cytosolic DNA fragments, which typically originate from viral infections or, e.g. in case of cell death, DNA damage (15–17). These DNA fragments are recognized by cyclic GMP-AMP synthase (cGAS), which, upon activation, catalyzes the synthesis of cyclic GMP-AMP (cGAMP) (18). This second messenger then triggers the phosphorylation of STING and TBK1, leading to the upregulation of interferon-stimulated genes (ISGs) and the subsequent recruitment and activation of immune cells (19, 20). This pathway is a key driver in shifting the TIME toward a robust anti-tumor state (21). Various cancer therapies, such as radiotherapy and specific chemotherapies, activate the STING pathway by inducing the accumulation of aberrant cytosolic DNA (15, 22–24). Although NIPP is well-documented for its cytotoxic effects on breast cancer cells, including the induction of DNA damage, its potential role in triggering STING activation and reprogramming the immune landscape remains largely uninvestigated.

Here, we explore the potential of direct and indirect plasma treatment in activating STING pathway and its ability in mediating immune response, potentially bridging NIPP’s cytotoxic and immunomodulatory effects in breast cancer therapy.

## 2 Materials and methods

### 2.1 Cell culture

The human breast cancer cell line MDA-MB-231 and patient-derived organoids (PDOs) from patients with TNBC were used as *in-vitro* tumor models, the human monocyte cell line THP-1 (kindly provided by Prof. Dr. Schenke-Layland Lab at the Department of Medical Technologies and Regenerative Medicine, at the University of Tübingen) as cellular immune compound, respectively. Four distinct culture media—M1, M2, M3, and M4—were utilized in these experiments. M1 consisted of Dulbecco’s Modified Eagle’s Medium (DMEM) (Gibco, Paisley, UK) supplemented with 10% fetal calf serum (FCS) and 1% penicillin-streptomycin (Gibco). M2 comprised DMEM without additional supplements, whereas M3 referred to RPMI 1640 GlutaMAX™ medium supplemented with 10% FCS and 1% penicillin-streptomycin. M4, specifically used for organoid cultivation, consisted of Advanced DMEM/F-12 supplemented with 1× GlutaMAX™ (Gibco), 10 mM HEPES (Gibco), and 1% penicillin-streptomycin (Gibco). M1 and M3 media were used for the seeding and cultivation of MDA-MB-231 and THP-1 cells, respectively. Cells were maintained in a humidified incubator at 37°C with 5% CO_2_. The culture medium was refreshed every 2–4 days, and cells reaching 70–80% confluence were detached using 0.05% trypsin-EDTA (Gibco) for passaging. Organoids were derived from the pleural effusion of a patient with metastatic TNBC treated at the University Women’s Hospital in Tübingen, following written informed consent (**Table 1**). The use of human donor cells was approved by the ethics committee of the medical faculty at the Eberhard Karl’s University Tübingen (495/2018BO2). Organoids were generated and cultured as previously described (25). Briefly, they were embedded in Basement Membrane Extract (BME, Bio-Techne) as single cells and seeded in 48-well plates at a density of 15,000 cells per well. Each well contained a 20 µL cell suspension-BME droplet at a ratio of 30% cell suspension to 70% BME. For passaging, organoids were washed and harvested in cold PBS/Y (PBS supplemented with 10 µM ROCK inhibitor Y-27632). Then, they were centrifuged, resuspended in TrypLE (Thermo Scientific) and incubated at 37°C in a water bath for 5 minutes. Following another centrifugation step, the supernatant was removed, and the pellet was resuspended in M4 medium for either reseeding or cryopreservation.

**Table 1:** Patient characteristics.

PDO-ID	Primary diagnosis						
	Histology	ER	PR	HER2	Clinical subtype	Treatment regimen	pCR
	NST	0%	20%	3+	HER2	E + C + PTX+H+P	No
#123A	Metastatic disease						
	DFI	ER	PR	HER2	Clinical subtype	Treatment regimen	Source
	30 months	0%	0%	2+; FISH: neg.	TNBC	nab-PTX + Pembro	PE

neg., negative; pos., positive; NST, No Special Type; FISH, Fluorescence *In Situ* Hybridization; pCR, pathologic Complete Response; E, Epirubicin; C, Cyclophosphamide; PTX, Paclitaxel; H, Trastuzumab; P, Pertuzumab; nab-PTX, nab-Paclitaxel; Pembro, Pembrolizumab; DFI, Disease Free Interval; PE, Pleural Effusion.

### 2.2 THP-1 differentiation and macrophages polarization

THP-1 cells were seeded in a 6-well plate at a density of 2 × 10^6^ cells per well and incubated in M3 medium with 80 nM phorbol 12-myristate 13-acetate (PMA; Sigma) for 48 hours to induce differentiation. This was followed by an additional 48-hour resting period in M3 medium without PMA to allow for macrophage maturation. The resulting THP-1-derived macrophages (M0) were then polarized by incubation with conditioned media for 24 hours. The conditioned medium was prepared as follows: MDA-MB-231 cells were treated with plasma, either APD or PTS, in the presence or absence of the STING inhibitor H-151, alongside a control group. Six hours after treatment, the existing medium was replaced with fresh M1 medium, and the cells were further cultured for an additional 48 hours. The supernatants from these five conditions were then collected and used as conditioned media for subsequent experiments.

### 2.3 NIPP device and treatment

The electrosurgical device VIO^®^ 3/APC 3 (Erbe Elektromedizin, Tübingen, Germany) was used to generate NIPP/APD in specifically tailored settings (Argon gas flow: 1.6 L/min; precise APC mode, effect 1). This mode delivers a continuous low-energy output, with an automatic precise adjustment of effect within a probe-to-tissue distance of up to 5 mm. For direct treatment, the previous medium was replaced by M2 medium. Cells covered with M2 medium were directly exposed to NIPP. For indirect treatment (PTS), M2 medium was firstly added into empty well and exposed to NIPP. Subsequently, the plasma-treated M2 medium (PTS) was transferred into the wells containing the pre-seeded cells, completely replacing the previous medium with PTS.

### 2.4 RONS measurement

The concentration of RONS was measured using customary semiquantitative test strips “Quantofix Peroxide 25”, “Quantofix Peroxide 100” and “Quantofix Nitrat/Nitrit 100” (Macherey-Nagel) respectively. The colorimetric test strips adapt to a certain coloring in the presence of specific reactive species. For measurements, 1 mL of DMEM without supplements was exposed to NIPP for indicated energy, followed by immediate immersion of the test strips for 1 second and read out using QUANTOFIX Relax (Macherey-Nagel), which provided numerical values based on internal calibration.

### 2.5 Cell viability assay

Cell viability of MDA-MB-231 cells was assessed using the colorimetric MTS assay. Cells were seeded in a 24-well plate at a density of 8 × 10^4^ cells per well and incubated for 24 hours. Following plasma treatment, the total volume of the culture medium was adjusted to 500 µL for further incubation. After the designated incubation period, the medium was replaced with 300 µL of M1 medium supplemented with 30 µL of MTS reagent. The plates were then incubated for 2 hours. The absorbance was measured at 490 nm using a Varioskan™ LUX multimode microplate reader (Thermo Scientific).

Organoid cell viability was assessed using the CellTiter-Glo^®^ 3D Cell Viability Assay. Organoids were cultured for 11 to 14 days and subsequently treated with PTS. On the second day post-treatment, the culture medium was removed, and 200 µL of fresh M1 medium along with 200 µL of CellTiter-Glo^®^ reagent was added to each well. The contents were vigorously mixed for 5 minutes and then incubated at room temperature for 25 minutes. Luminescence was measured using Varioskan™ LUX multimode microplate reader (Thermo Scientific) with an integration time of 1 second per well.

### 2.6 Apoptosis analysis

MDA-MB-231 cells were seeded in a 24-well plate and incubated in M1 medium overnight. The following day, cells were subjected to gas flow (negative control), plasma treatment. After 6 hours of incubation, the culture medium was replaced with 200 µL of M1 medium and 200 µL of 2X RealTime-Glo™ Annexin V Apoptosis Detection Reagent (Promega). Apoptosis was measured every 8 hours for a total of 48 hours. Time point 0 referred to the first apoptosis measurement, taken 6 hours after plasma treatment. The detection reagent was prepared according to the manufacturer’s instructions by sequentially adding 1,000X Annexin NanoBiT^®^ Substrate, 1,000X CaCl_2_, 1,000X Annexin V-SmBiT, and 1,000X Annexin V-LgBiT into prewarmed M1 medium. Luminescence was measured with an integration time of 1 second per well.

### 2.7 Western blot analysis

Proteins were extracted from tumor cells using RIPA lysis buffer (Thermo Fisher Scientific) mixed with protease inhibitors (MedChemExpress) and phosphatase inhibitors (Sigma Aldrich). Protein concentrations were quantified using the BCA protein assay kit (Thermo Fisher Scientific). Then, 100 µg of total protein was loaded per lane. Proteins were separated by 10% SDS-PAGE and transferred from gel to a nitrocellulose membrane (Sigma Aldrich) using Trans-Blot Turbo transfer system (Bio-Rad).

Membranes were blocked with 5% bovine serum albumin (BSA) in 0.1% TBST (0.1% Tween 20) and subsequently incubated overnight at 4°C with primary antibodies diluted in 0.1% TBST supplemented with 5% BSA. The following primary antibodies were used: anti-STING (#13647, 1:1,000 dilution, Cell Signaling Technology), anti-Phospho-STING (Ser366) (#19781, 1:1,000 dilution, Cell Signaling Technology), anti-Phospho-TBK1/NAK (Ser172) (#5483, 1:1,000 dilution, Cell Signaling Technology), anti-TBK1/NAK (#3504, 1:1,000 dilution, Cell Signaling Technology), anti-Phospho-Histone H2A.X (Ser139) (#9718, 1:1,000 dilution, Cell Signaling Technology), and anti-β-actin (#66009-1-Ig, 1:20,000 dilution, Proteintech).

On the next day, membranes were incubated for 1 hour at room temperature with anti-rabbit (#7074, 1:1,000 dilution, Signaling Technology) or anti-mouse (#7076, 1:1,000 dilution, Signaling Technology) secondary antibody conjugated to horseradish peroxidase diluted in 0.1% TBST supplemented with 5% BSA. Immunoblots were visualized using the ECL Western Blotting Substrate (Thermo Fisher Scientific) and imaged with the iBright CL1000 imaging system (Invitrogen).

### 2.8 RNA isolation, cDNA synthesis and RT-qPCR

Total RNA was extracted using 1 mL of TRIzol reagent (Invitrogen) per sample, following the manufacturer’s instructions. Complementary DNA (cDNA) synthesis was performed using M-MLV Reverse Transcriptase (Promega) according to the manufacturer’s protocol, utilizing the T100 Thermal Cycler (Bio-Rad). Quantitative real-time PCR (qRT-PCR) was conducted using the QuantStudio™ 3 Real-Time PCR System (Applied Biosystems) with PowerUp SYBR Green Master Mix (Applied Biosystems) and 2.5 µL of cDNA was used for each 20 µL reaction system. The qPCR program was 95°C for 2 minutes, followed by 40 cycles of 95°C for 15 seconds, 59°C for 15 seconds and 72°C for 1 minute. GAPDH was used as an internal reference control. The 2^−ΔΔCT^ method was used to calculate the relative mRNA level.

The following primers were used:

GAPDH forward, 5’- GTCTCCTCTGACTTCAACAGCG -3’.
GAPDH reverse, 5’- ACCACCCTGTTGCTGTAGCCAA -3’.
CCL5 forward, 5’- CCTGCTGCTTTGCCTACATTGC -3’.
CCL5 reverse, 5’- ACACACTTGGCGGTTCTTTCGG -3’.
IFN-β forward, 5’- CTTGGATTCCTACAAAGAAGCAGC -3’.
IFN-β reverse, 5’- TCCTCCTTCTGGAACTGCTGCA -3’.
IFIT1 forward, 5’- GCCTTGCTGAAGTGTGGAGGAA -3’.
IFIT1 reverse, 5’- ATCCAGGCGATAGGCAGAGATC -3’.
OAS1 forward, 5’- AGGAAAGGTGCTTCCGAGGTAG -3’.
OAS1 reverse, 5’- GGACTGAGGAAGACAACCAGGT -3’.
ISG15 forward, 5’- CTCTGAGCATCCTGGTGAGGAA -3’.
ISG15 reverse, 5’- AAGGTCAGCCAGAACAGGTCGT -3’.
CXCL10 forward, 5’- GGTGAGAAGAGATGTCTGAATCC -3’.
CXCL10 reverse, 5’- GTCCATCCTTGGAAGCACTGCA -3’.

### 2.9 Flow cytometry

Adherent macrophages were detached using 0.05% trypsin-EDTA. The trypsinization process was halted by adding of M3 medium. The harvested cells were washed with PBS and resuspended in ice-cold FACS buffer (autoMACS^®^ Rinsing Solution supplemented with 0.5% bovine serum albumin) at a concentration of 1 × 10^7^ cells/mL. A 100 µL aliquot of the cell suspension was transferred to a universal tube and incubated with 2 µL of antibody in the dark at 4°C for 10 minutes. The antibodies used included FITC-labeled anti-CD11b (#AB_2536479, 1:50 dilution, Miltenyi Biotec) and APD-labeled anti-CD80 (#AB_1283666, 1:50 dilution, Miltenyi Biotec). Flow cytometric analysis was performed using a FACSCanto™ II flow cytometer, and data were analyzed with FlowJo 10.8.1 software (Becton Dickinson).

### 2.10 Statistical analysis

SPSS statistical software was used for data analysis. Statistical analyses were performed using a one-way ANOVA or Student’s t-test. A p-value of < 0.05 was considered statistically significant. Values are presented as mean ± standard error of mean (SEM). Each experiment was performed in at least three independent replicates.

## 3 Results

### 3.1 NIPP generates RONS and reduces MDA-MB-231 cell viability in a dose-dependent manner

**Figure 1:**
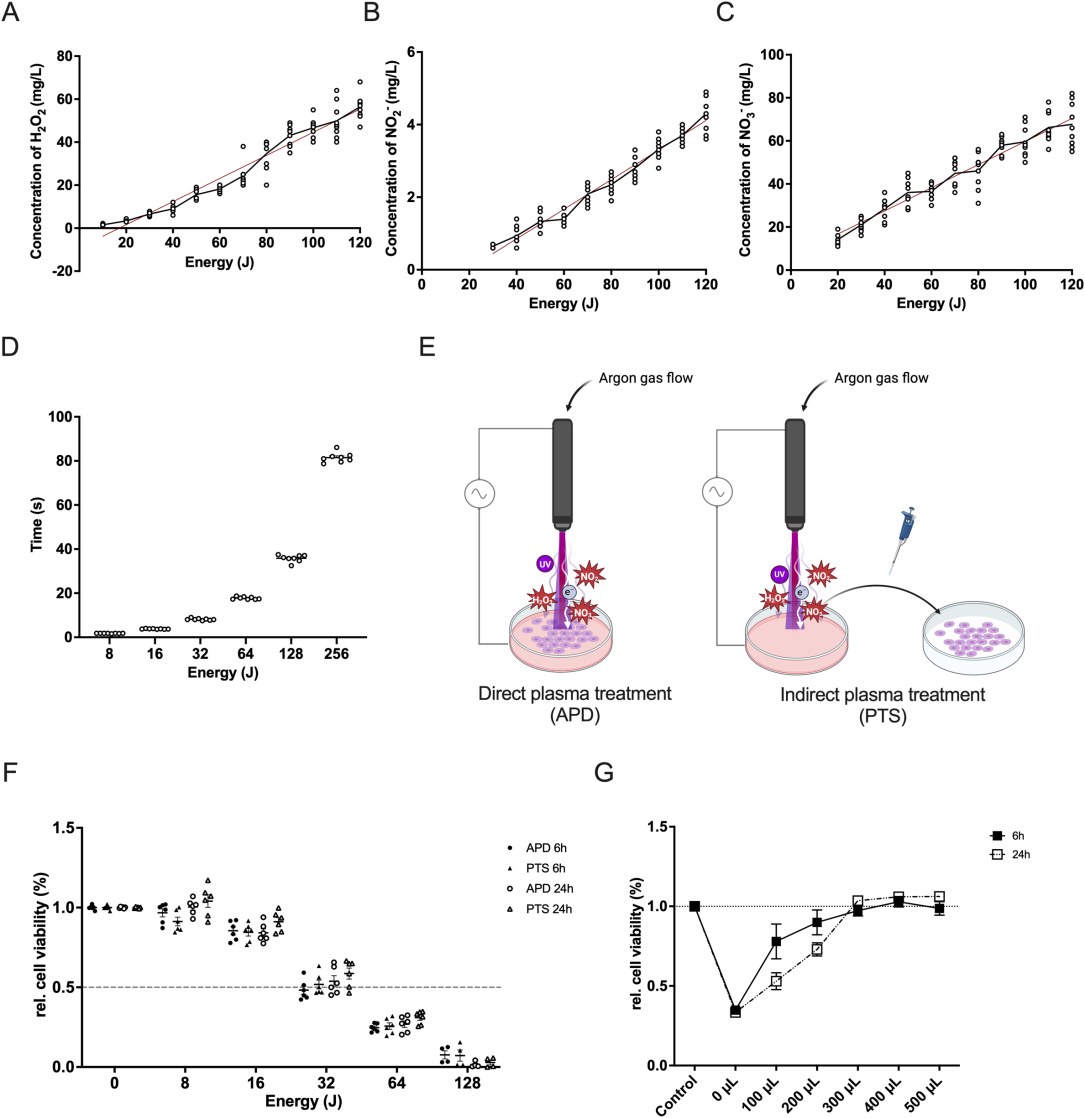
Characterization of NIPP and its cytotoxic effect in triple-negative breast cancer cells. **(A–C)** Correlation between plasma energy and the production of H_2_O_2_ **(A)**, NO_2_^-^ **(B)**, and NO_3_^-^ **(C)**. Data points represent measured values. The regression line is shown as a red solid line. **(D)** Time required for the NIPP device to generate specific plasma energy. **(E)** Schematic representation of direct plasma treatment (APD) and indirect plasma treatment (PTS) using NIPP. **(F)** Relative cell viability of MDA-MB-231 cells after 6 hours or 24 hours of incubation following APD, PTS, and control treatments. Cells were covered with 500 µL of M2 medium during APD treatment. **(G)** Relative cell viability of MDA-MB-231 cells after 6 hours or 24 hours of incubation following 36 seconds of gas flow intervention and control treatment. Cells were covered with various volumes of M2 medium (0 µL, 100 µL, 200 µL, 300 µL, 400 µL, and 500 µL) and topped up to 500 µL during incubation. Statistical comparison was performed with paired Student’s t-tests. (mean ± SEM; *p < 0.05). Panel **(E)** was created in BioRender. Weiss, M. (2025) https://BioRender.com/9zkxpqs.

During plasma treatment, a variety of reactive molecules are generated, with RONS being the most significant due to their extensive biological effects. Results demonstrated that plasma treatment led to a linear increase in the concentrations of H_2_O_2_, NO_2_^-^, and NO_3_^-^ with increasing energy, as shown by the calculated rates of production (0.54, 0.04, and 0.54, respectively) and strong correlations (R² values of 0.93, 0.94, and 0.87, respectively) (**Figures 1A–C**). This suggests that higher energy input results in increased RONS concentrations, which in turn amplify the biological effects on the target. The plasma device generates higher energy by extending the exposure time. By recording both the exposure time and the final energy, we observed a proportional increase in energy over time (**Figure 1D**).

To investigate the cytotoxic effect of plasma treatment, MDA-MB-231 cells were treated with either APD or PTS. During APD treatment, cells were covered with 500 µL of M2 medium (**Figure 1E**). Both APD and PTS treatments exhibited similar cytotoxic effects after both 6 and 24 hours of incubation (**Figure 1F**). There was no significant difference between APD and PTS, suggesting that excessive covering medium may have attenuated the direct plasma treatment effect. To address this, we reduced the volume of the covering medium. To assess whether gas flow alone has an effect on cell viability, MDA-MB-231 cells were exposed to 36 seconds of direct argon gas flow without plasma ignition. This exposure time was chosen as it was sufficient for the plasma device to generate 128 Joules of energy, enough to induce significant cell death (**Figure 1F**). Cell viability significantly decreased without covering medium (**Figure 1G**). As medium volume increased, viability recovery occurred, with no further effect when the volume exceeded 300 µL.

### 3.2 APD more effectively reduces cell viability and induces apoptosis in MDA-MB-231 cells compared to PTS

**Figure 2:**
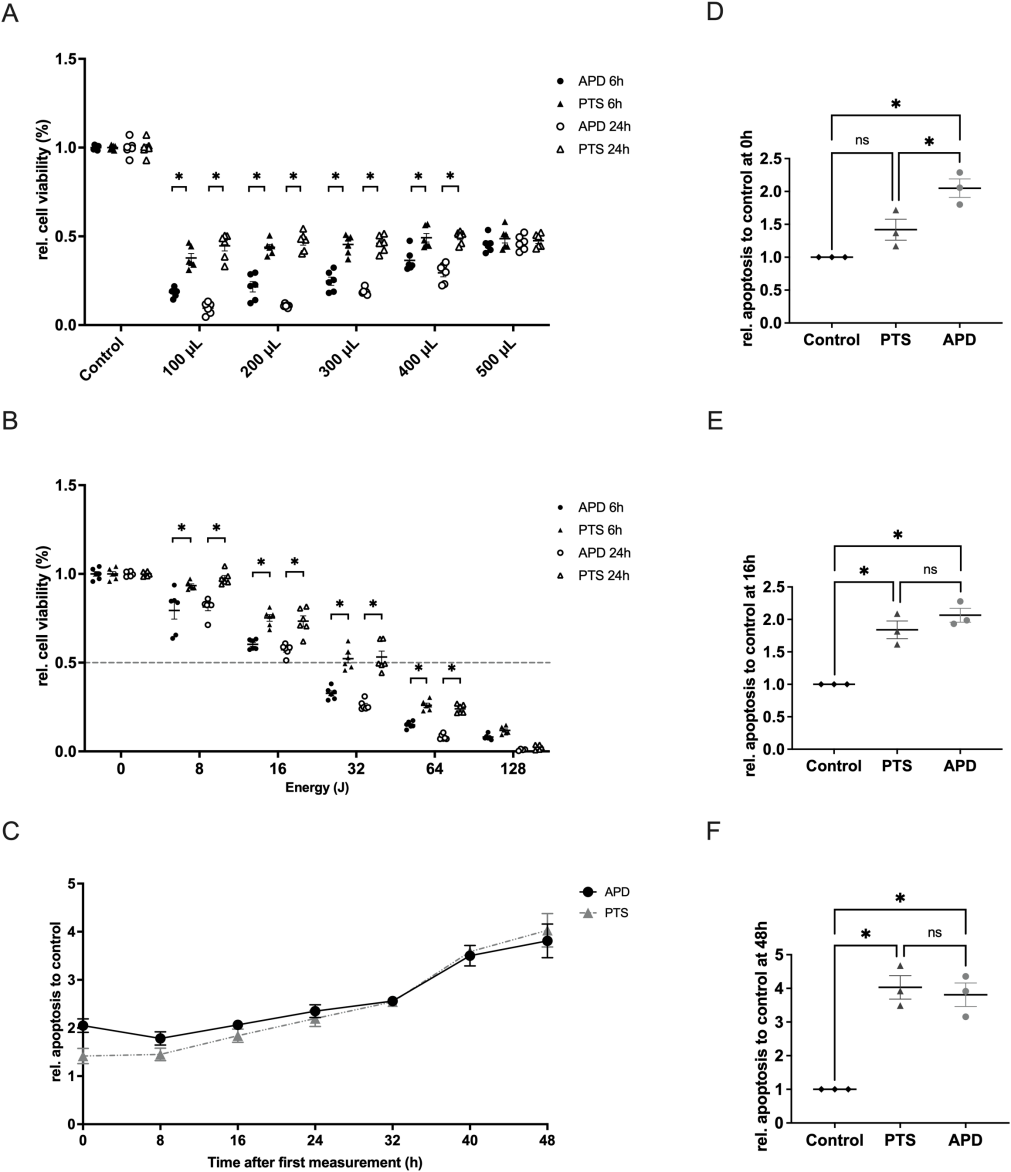
Characterization of the cellular effects of APD and PTS on MDA-MB-231 cells when the protective effect of culture medium was excluded. **(A)** Relative cell viability of MDA-MB-231 cells after 6 hours or 24 hours of incubation following 32 Joules of APD, PTS, or control treatments. Cells were covered with various volumes of M2 medium (0 µL, 100 µL, 200 µL, 300 µL, 400 µL, and 500 µL) and topped up to 500 µL during incubation. **(B)** Relative cell viability of MDA-MB-231 cells after 6 hours or 24 hours of incubation following APD, PTS, or control treatments. Cells were covered with 400 µL of M2 medium during treatment. **(C)** Relative apoptosis levels of MDA-MB-231 cells within 48 hours after 6 hours of incubation following APD, PTS, or control treatments. Time point 0 h refers to the time immediately after the 6 hours incubation following treatment. **(D–F)** Comparison of apoptosis levels among the APD, PTS, and control groups at the 0 h **(D)**, 16 h **(E)**, and 48 h **(F)** time points. Statistical comparisons were performed using the Student’s t-test for two-group comparisons or one-way ANOVA for comparisons across multiple groups. (mean ± SEM; *p < 0.05).

To compare the cytotoxic effects of APD and PTS with decreasing medium volume, various volumes of M2 medium (100 µL to 500 µL) were used, all treated with 32 Joules of plasma energy. After treatment, the medium was adjusted to 500 µL. Results showed that under the same energy conditions, APD’s efficacy increased as covering medium volume decreased (p < 0.05), while PTS effect remained stable from 500 µL to 100 µL (**Figure 2A**). The reduction in medium volume helped minimize the neutralizing effect of the covering medium and excluded the influence of gas flow. Results showed that APD was more effective than PTS in decreasing the cell viability (**Figure 2B**). Apoptosis analysis revealed that both APD and PTS induced apoptosis, with APD showing an early onset of apoptosis at the 0-hour time point after treatment (**Figures 2C–F**).

### 3.3 APD and PTS induce STING pathway activation in TNBC cells

**Figure 3:**
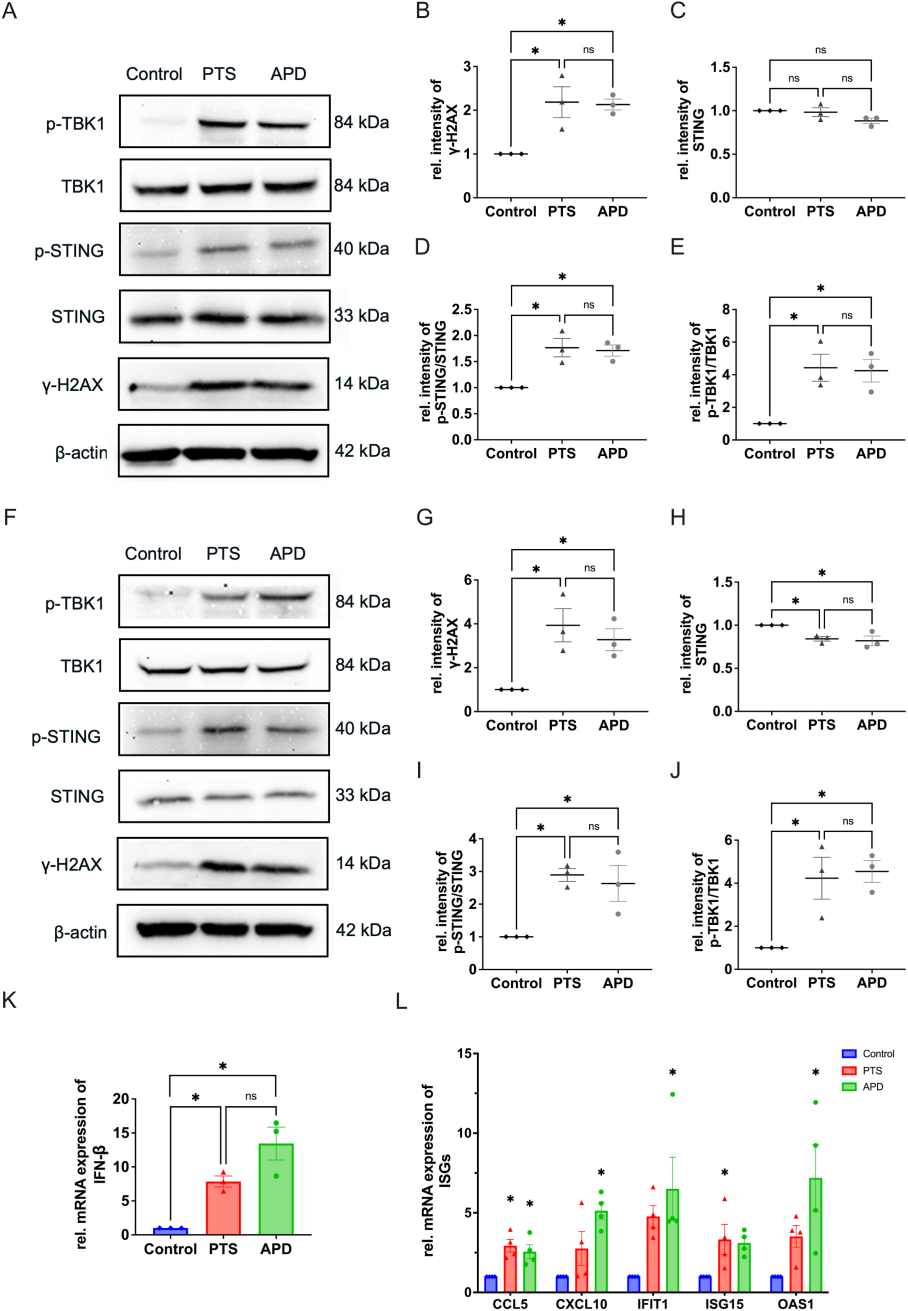
APD and PTS activate the STING pathway in MDA-MB-231 cells. **(A)** Representative immunoblots showing γ-H2AX, total STING, p-STING, TBK1, and p-TBK1 expression in MDA-MB-231 cells after 6 hours of incubation following APD, PTS, or control treatment. β-actin was used as a loading control. **(B–E)** Quantification of relative protein expression levels: γ-H2AX **(B)**, total STING **(C)**, p-STING/STING ratio **(D)**, and p-TBK1/TBK1 ratio **(E)**. **(F)** Representative immunoblots of γ-H2AX, total STING, p-STING, TBK1, and p-TBK1 expression in MDA-MB-231 cells after 24 hours of incubation following APD, PTS, or control treatment. β-actin was used as a loading control. **(G–J)** Quantification of relative protein expression levels: γ-H2AX **(G)**, total STING **(H)**, p-STING/STING ratio **(I)**, and p-TBK1/TBK1 ratio **(J)**. **(K)** RT-qPCR analysis of the gene expression of IFN-β in MDA-MB-231 cells treated with APD, PTS, or control and incubated for 6 hours. **(L)** RT-qPCR analysis of the gene expression of CCL5, CXCL10, IFIT1, ISG15, and OAS1 in MDA-MB-231 cells treated with APD, PTS, or control and incubated for 6 hours. GAPDH was used as a reference gene. Statistical comparisons were conducted using one-way ANOVA for parametric data and the Kruskal-Wallis test for non-parametric data. (mean ± SEM; *p < 0.05).

We next investigated the impact of APD and PTS on the activation of the STING pathway at both the gene and protein expression levels. Since many therapeutics activate STING by inducing DNA damage, we first examined γ-H2AX, a marker of DNA double-strand breaks. Western blot analysis showed a significant increase in γ-H2AX expression following both APD and PTS treatments compared to the control group (**Figures 3A, B**). These aberrant DNA fragments are subsequently recognized by cGAS, which catalyzes the synthesis of 2′3′-cGAMP. This cyclic dinucleotide acts as a secondary messenger, binding to STING and initiating the signaling pathway (20). Western blotting showed an enhanced STING activation, as indicated by a higher p-STING/STING ratio in both APD- and PTS-treated cells (**Figures 3A, D**). As a key downstream effector of the STING pathway, p-TBK1 expression was further analyzed by western blotting in MDA-MB-231 cells following both direct and indirect plasma treatments. The results revealed a significant increase in p-TBK1 levels, while total TBK1 expression remained unchanged, leading to a markedly higher p-TBK1/TBK1 ratio compared to the control (p < 0.05) (**Figures 3A, E**).

To assess the persistence of these effects, we further examined protein expression at 24 hours post-treatment. Both direct (APD) and indirect (PTS) plasma treatments continued to induce DNA damage and sustained activation of the STING-TBK1 axis as shown by the increased expression level of γ-H2AX, as well as the p-STING/STING ratio and p-TBK1/TBK1 ratio at this timepoint (**Figures 3F–J**). After prolonged incubation, STING expression decreased in APD- and PTS-treated cells (p < 0.05), suggesting its degradation following signal transduction, consistent with findings from previous studies (26, 27) (**Figures 3C, H**).

The p-TBK1 protein is an important modulator for the IFN-β production. As a downstream event of STING activation, the transcription of IFN-β plays a crucial role in immune signaling. To further investigate the IFN-β signal pathway activation by APD and PTS, we used RT-PCR to determine the expression level of genes of IFN-β in MAD-MB-231 cells. The results revealed a significant upregulation of IFN-β expression following APD and PTS treatments (**Figure 3K**). Furthermore, key ISGs, including CCL5, CXCL10, IFIT1, ISG15, and OAS1, were also upregulated, indicating an immune response induced by plasma treatment (**Figure 3L**).

Taken together, plasma treatment effectively induced DNA double-strand breaks and subsequent STING pathway activation.

### 3.4 M1 polarization of THP-1-derived macrophages induced by APD and PTS is associated with STING activation

**Figure 4:**
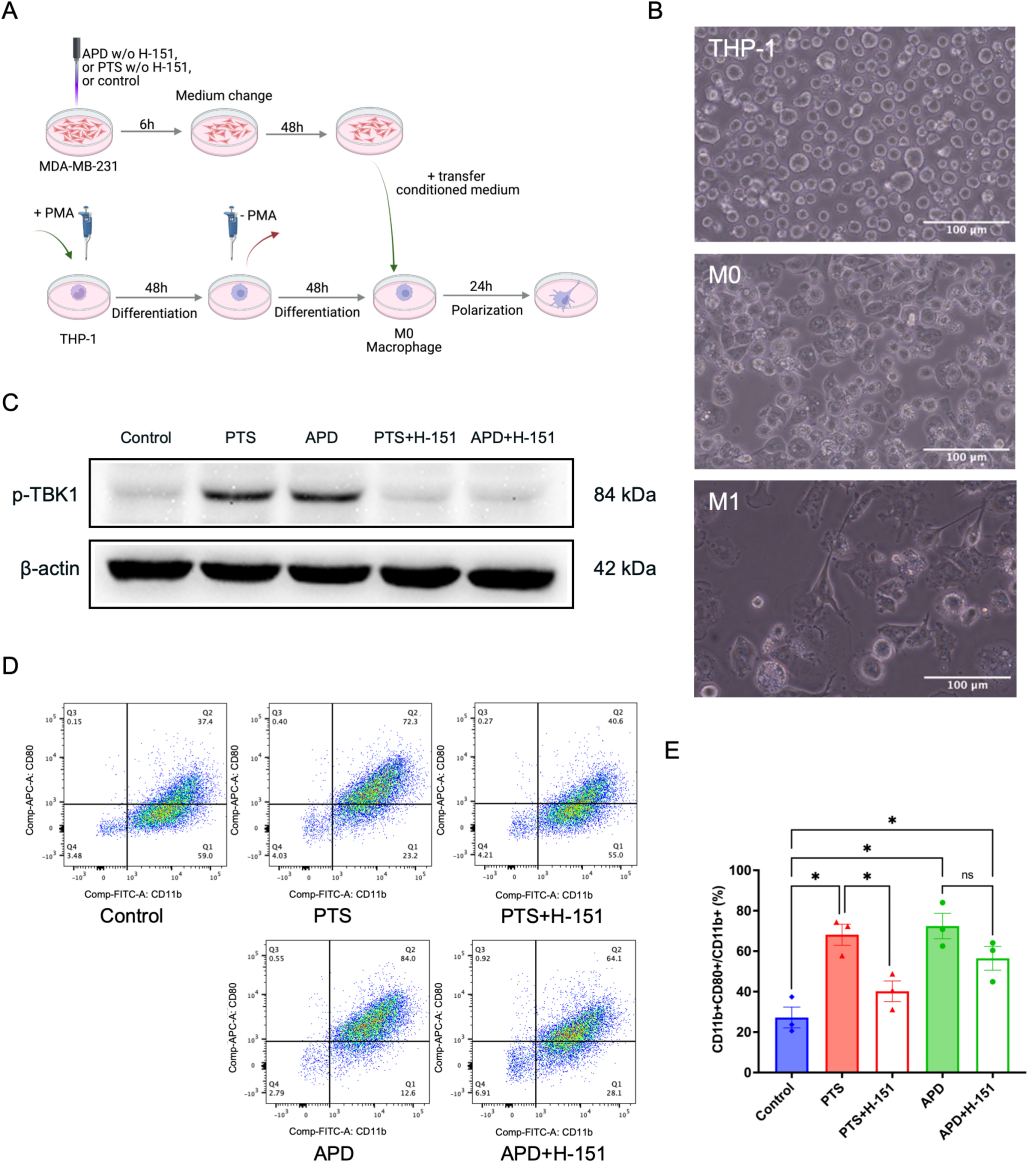
APD and PTS polarize THP-1 derived macrophages into an M1 phenotype in a STING-dependent manner. **(A)** Schematic of conditioned media experiments using macrophages derived from THP-1 cells. **(B)** Representative brightfield images of THP-1, M0, and M1 macrophages. Scale bar: 100 μm. **(C)** Representative immunoblots showing p-TBK1 expression in MDA-MB-231 cells following APD, PTS, or control treatment, with or without the STING inhibitor H-151. β-actin was used as a loading control. **(D)** Representative flow cytometry plots of macrophages exposed to various conditioned media derived from MDA-MB-231 cells treated with APD, PTS, or control, with or without the STING inhibitor H-151. Subpopulations were gated based on CD11b and CD80 expression, with M1 macrophages identified as CD11b+ CD80+. **(E)** The respective statistical analysis of the flowcytometry results. Statistical comparison was performed with one-way ANOVA. (mean ± SEM; *p < 0.05). Panel **(A)** was created in BioRender. Weiss, M. (2025) https://BioRender.com/a98tm0v.

Within the tumor microenvironment, cancer cells exist in a complex network of interactions with various cell types, including fibroblasts, macrophages, and lymphocytes. To investigate how plasma-treated cancer cells influence surrounding immune cells and determine whether this effect is mediated through the STING pathway, we utilized the monocyte cell line THP-1. These cells were differentiated into M0 macrophages by incubation with 80 nM PMA for 48 hours (**Figure 4A**), a concentration and duration previously demonstrated to be effective (28). Morphologically, THP-1 cells appeared as round cells in suspension, while differentiated into M0 macrophages the adherent cells showed a round and partly donut-like shape. In contrast, after co-culture with conditioned media, activated macrophages exhibited an M1 phenotype, characterized by an elongated, amoeboid shape and numerous fibrillary cytoplasmic structures (**Figure 4B**).

To determine whether plasma-induced M1 polarization was STING-dependent, we employed the STING inhibitor H-151, which blocks STING activation by preventing its palmitoylation at the Golgi apparatus, thereby suppressing TBK1 activation (29). Western blot analysis revealed a reduction in p-TBK1 levels in APD- and PTS-treated samples when H-151 was present, confirming successful STING inhibition (**Figure 4C**). Flow cytometry analysis further demonstrated a significant increase in the percentage of CD11b^+^CD80^+^ M1 macrophages in the APD and PTS treatment groups compared to the control (**Figures 4D, E**). Notably, the addition of H-151 to MDA-MB-231 cells prior to PTS treatment significantly reduced this M1 polarization effect (p < 0.05). Furthermore, a numerical reduction was observed in the APD + H-151 group compared to the APD-only group.

**Figure 5:**
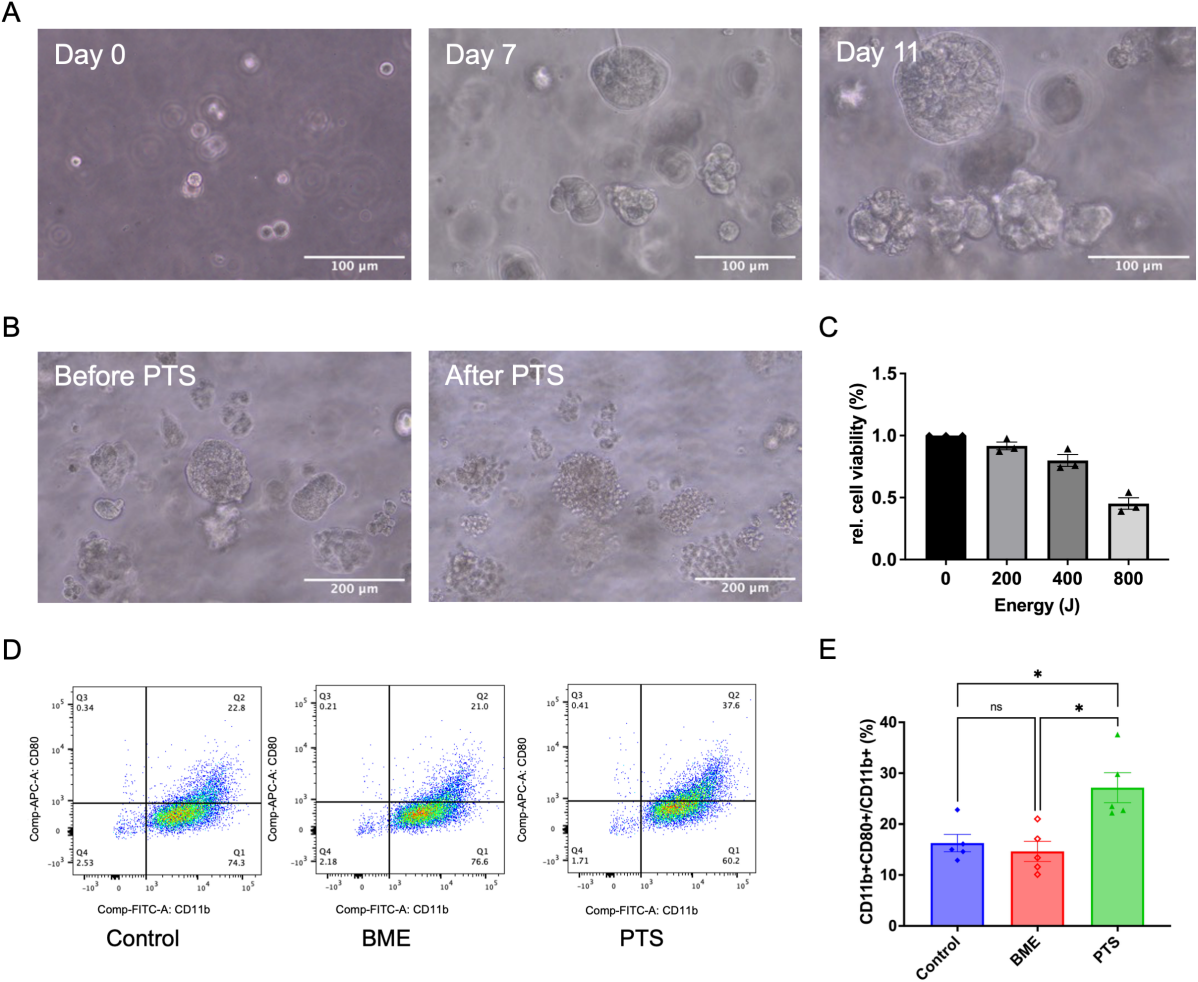
PTS induces M1 polarization of THP-1 derived macrophages in a TNBC patient-derived organoid model. **(A)** Representative brightfield images showing the formation of TNBC patient-derived organoids on day 0, day 7, and day 11. Scale bar: 100 μm. **(B)** Representative brightfield images of TNBC patient-derived organoids before and after PTS treatment. Scale bar: 200 μm. **(C)** Relative cell viability of TNBC patient-derived organoids after 24 h of incubation following PTS treatment, assessed using the CellTiter-Glo^®^ 3D Cell Viability Assay. **(D)** Representative flow cytometry plots of macrophages exposed to conditioned media derived from TNBC patient-derived organoids treated with PTS or control. ‘BME’ refers to a subgroup with the BME biomaterial scaffold but without cells. M1 macrophages were identified as CD11b+ CD80+. **(E)** The respective statistical analysis of the flowcytometry results. Statistical comparison was performed with one-way ANOVA. (mean ± SEM; *p < 0.05).

Following the demonstration of plasma’s immunomodulatory effects using a commercially available breast cancer cell line, we employed patient-derived primary breast cancer organoids to further validate these findings, as organoids better mimic the physiological relevance of actual tumors compared to cell lines. The organoids were generated from pleural effusion obtained from a female patient with triple-negative breast cancer. After 10 to 14 days of incubation, the organoids formed dense and irregular structures (**Figure 5A**). The present data show that PTS induced M1 polarization in a STING-dependent manner (**Figure 4E**). Additionally, PTS was more manageable compared to APD in small wells. Once the organoid cultures were successfully established, plasma treatment effects were evaluated using PTS. Brightfield imaging revealed structural disruption of the organoids and extensive cell death (**Figure 5B**). Additionally, viability assays confirmed a dose-dependent reduction in organoid viability following PTS treatment (**Figure 5C**). The polarization of THP-1-derived macrophages was induced by transferring conditioned medium from the organoids, following the same protocol as shown in **Figure 4A**. Since the basement membrane extract (BME), used as a scaffold for organoid culture, is composed of biomaterial, it may have potential effects on M1 polarization when exposed to plasma. To account for this, an additional control group containing only the BME scaffold (without cells) was included. After plasma treatment, the mean ratio of CD11b^+^CD80^+^/CD11b^+^ cells increased, indicating M1 polarization. Importantly, this increase was not attributed to the plasma-treated BME alone (**Figures 5D, E**).

## 4 Discussion

**Figure 6:**
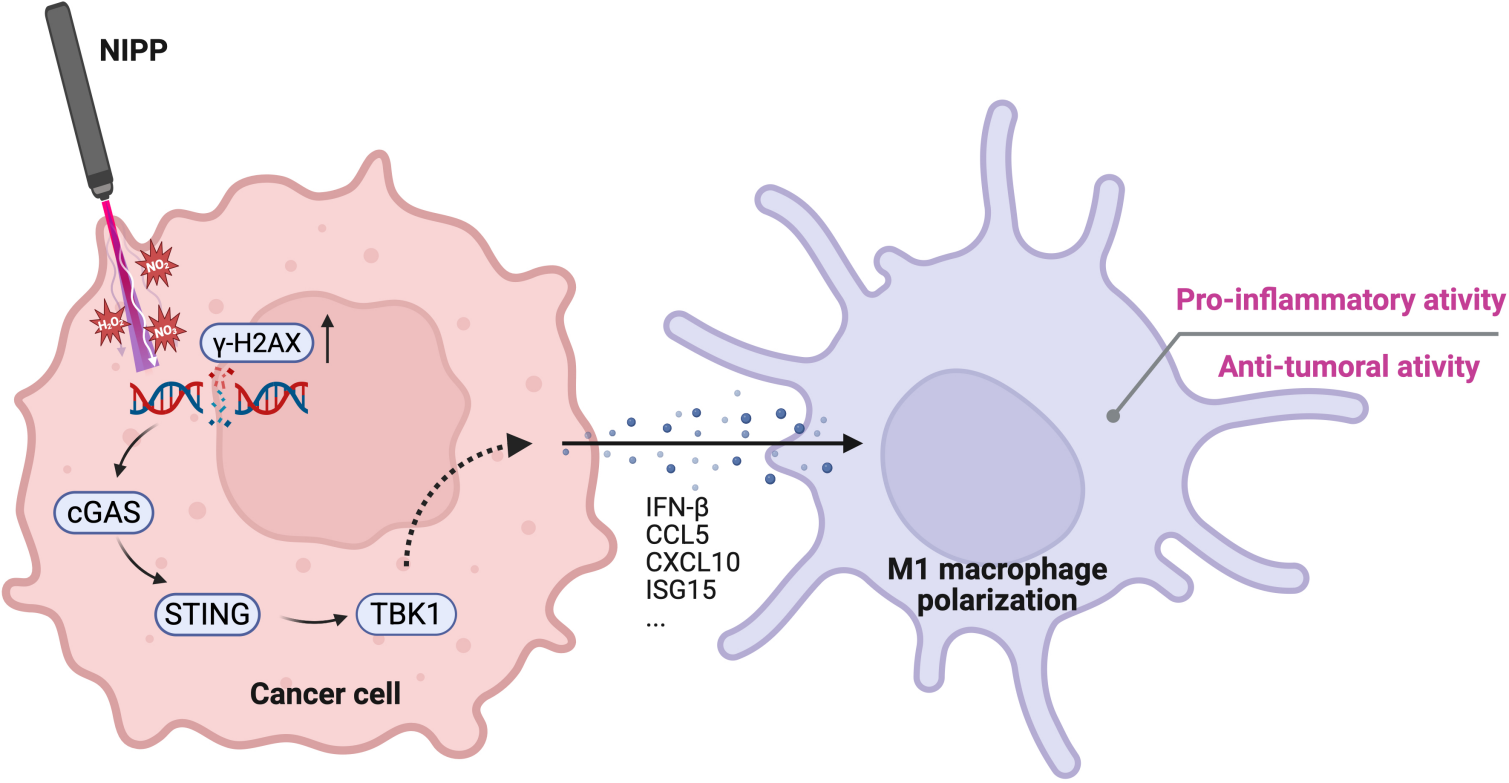
Proposed mechanism of NIPP-induced immune activation. NIPP treatment generates RONS, leading to DNA damage in cancer cells, as indicated by elevated γ-H2AX expression. The aberrant cytosolic DNA fragments are detected by cGAS, which subsequently activates the STING pathway. This signaling cascade promotes the polarization of THP-1 derived macrophages toward a pro-inflammatory M1 phenotype, contributing to the anti-tumor immune response. Created in BioRender. Weiss, M. (2025) https://BioRender.com/mxg10am.

Although TNBC is a highly aggressive breast cancer subtype that lacks the classical molecular pattern for targeted therapies, it exhibits a higher mutation burden and greater immune cell infiltration compared to other breast cancer subtypes (3, 4). These characteristics make therapeutic strategies aimed at modulating the TIME particularly promising (5, 6). In this study, we observed that NIPP significantly reduced the viability of TNBC cell lines as well as patient-derived organoids. Furthermore, NIPP treatment induced a shift in the TIME toward a pro-inflammatory state, and this transformation was associated with the activation of the STING pathway (**Figure 6**). This finding highlights the dual role of NIPP in TNBC treatment, bridging its cytotoxic and immunomodulatory effects.

The cytotoxic effects of plasma have been well established; however, certain mechanistic details remain to be elucidated. In this study, we compared the direct and indirect application of plasma. After excluding the effect of gas flow, we observed that APD was more effective in reducing cell viability than PTS and was capable of inducing apoptosis at an earlier stage. To date, only a limited number of studies have compared the efficacy of direct and indirect plasma treatments with controversial results (30–33). For instance, Saadati et al. reported that direct plasma treatment resulted in higher levels of apoptosis, DNA damage, as well as a significant reduction in tumor growth in melanoma cells compared to indirect treatment (31). Similarly, Akbari et al. found that direct gas plasma exposure was slightly more potent than plasma-treated medium in primary human breast cancer tissue (33). These findings are consistent with our observations. In contrast, Gherardi et al. reported no significant differences between direct and indirect treatments on lymphoma cells (32). Notably, they used a dielectric barrier discharge (DBD) as plasma source, which makes the results difficult to compare. These findings underscore the importance of device-specific investigations when evaluating plasma-based therapies (34, 35).

In contrast to normal cells, cancer cells exhibit significantly higher mutation burdens and deficiencies in DNA repair genes. As a result, treatments that induce DNA damage can increase these genomic burdens, thereby increasing the likelihood of activating the STING pathway (23, 24). In this study, both APD and PTS treatments led to an increased expression of γ-H2AX, a marker of DNA double-strand breaks, followed by the activation of the STING pathway. This finding aligns with previous research on NIPP effects in different cancer entities as well as other reports on different breast cancer therapies, such as radiotherapy, paclitaxel, and Olaparib, which similarly activate the STING pathway through DNA damage (22–24, 36). Establishing a connection between plasma treatment and STING activation provides a mechanistic explanation for the potential synergistic effects of plasma therapy when combined with other treatments. For example, radiotherapy induces DNA double-strand breaks via ionizing radiation, triggering immunostimulatory effects mediated through the STING pathway (15, 37). Baird et al. demonstrated that the combination of a STING agonist with radiotherapy resulted in improved tumor control, prolonged overall survival, and enhanced adaptive immune responses compared to either treatment alone (38). Therefore, integrating plasma treatment with STING agonists could enhance the therapeutic efficacy of CAP, particularly in modulating TIME. This approach may be particularly beneficial for TNBC, where immune modulation represents a promising therapeutic strategy. Schultze-Rhonhof et al. recently reported about the robustness and low reactivity of NIPP-treated patient-derived tissue resident macrophages (39).

However, synergistic antitumor effects have been observed in several preclinical tumor models and clinical trials when checkpoint inhibitors are combined with CAP (40–44). For instance, Wang et al. reported that CAP treatment led to increased PD-L1 expression compared to the control group and demonstrated that adding PD-1 antibodies to CAP significantly enhanced tumor suppression and prolonged survival *in vivo* (40). Notably, PD-L1 expression is upregulated in response to STING activation, serving as negative feedback that dampens the immune response (45, 46). In the present study, STING pathway activation was observed following both APD and PTS treatments. Our findings on plasma-induced STING activation provide a rationale for the combination therapy of CAP and checkpoint inhibitors.

As downstream secondary events of STING activation, the upregulation of ISGs plays a critical role in modulating immunity. In this study, the transcriptional levels of key ISGs, including ISG15, OAS1, IFIT1, CCL5, and CXCL10, were significantly increased following APD and PTS treatments. CCL5 is involved in the recruitment of immune cells such as CD8+ T cells, CD4+ T cells, and M1 macrophages, enhancing anti-tumor immunity (47). IFIT1 has been reported to indirectly modulate immune responses, potentially by increasing CXCL10 expression, which facilitates lymphocyte chemotaxis (48, 49). Meanwhile, ISG15 and OAS1 play pivotal roles in innate immune defense, further underscoring the immunomodulatory potential of plasma treatment (50).

In this study, THP-1-derived M0 macrophages underwent pro-inflammatory M1 polarization after exposure to conditioned medium obtained from plasma-treated TNBC cells. These findings were consistently observed in both 2D cultures and patient-derived 3D models. Notably, following the post-plasma incubation period, the original medium containing RONS or other direct plasma-generated effectors was removed 6 hours after NIPP treatment. Consequently, the conditioned medium comprised only signaling molecules and effectors produced by plasma-treated cancer cells within 6 hours after NIPP exposure. This suggests that the observed pro-inflammatory activation of immune cells was driven by the cancer cells’ response to plasma treatment rather than the direct influence of plasma itself. Since THP-1-derived macrophages can undergo M1 polarization under direct influence of plasma (51). Using the STING inhibitor H-151, p-TBK-1 expression was reduced to nearly the level of the control group. Consistent with STING inhibition, the M1 polarization level of THP-1-derived M0 macrophages decreased.

Not all cancer cells possess a fully functional STING pathway and the loss of this pathway in cancer cells is a strategy to evade from host immune surveillance (21, 52, 53). Qiao et al. screened 22 human cancer cell lines across various cancer types and found that only 16 expressed STING at both the mRNA and protein levels (52). While the MDA-MB-231 cell line was confirmed to have an intact STING pathway, another TNBC cell line, MDA-MB-453, lacked STING expression (52). TNBC is a highly heterogeneous group of cancers. Therefore, the interpretation of our findings should be restricted to specific situation.

Interestingly, even when STING function is compromised within cancer cells, this pathway can still be activated through intercellular communication (54, 55). For instance, 2′3′-cGAMP can be exported from tumor cells and transferred to neighboring cells, thereby activating STING in both other cancer cells and immune cells (55, 56). Notably, 2′3′-cGAMP has been shown to promote M1 macrophage polarization (57, 58), which may explain why macrophages can still undergo M1 polarization even when the STING is inhibited.

Several limitations should be acknowledged in this study. The mechanistic characterization of STING pathway activation was performed exclusively in 2D cell cultures and could not be directly validated in the patient-derived organoid model due to fundamental technical constraints. Organoids contain heterogeneous cell populations compared to homogeneous cell lines, yield insufficient protein quantities, and the basement membrane extract scaffold interferes with protein extraction protocols. As a result, at least 500,000 cells are required for reliable Western blot analysis, whereas only 15,000 cells were seeded per well in the present study (59). Consequently, we focused on functional validation through macrophage polarization as the downstream endpoint of STING activation in organoids. Additionally, our immune response characterization was limited to THP-1-derived macrophages rather than primary immune cells, and we did not assess potential immunosuppressive feedback mechanisms or conduct systematic toxicity profiling in normal tissues. While patient-derived organoids provide superior clinical relevance compared to cell lines, the mechanistic insights from 2D cultures require validation through organoid-specific optimized protocols in future studies. These limitations highlight the need for continued method development to bridge 2D mechanistic findings with 3D patient-derived model validation.

## 5 Conclusions

Here, we report that NIPP effectively activate the STING pathway, a crucial bridge between innate and adaptive immunity. These molecular changes facilitated the M1 polarization of THP-1-derived macrophages in a STING-dependent manner. Our findings suggest that plasma therapy holds promise not only as a direct anticancer treatment but also as a strategy to enhance anti-tumor immune responses. Furthermore, this study highlights the predictive value of STING expression in assessing plasma-induced immune modulation and provides insights for future combination strategies involving plasma therapy.

## Data Availability

The original contributions presented in the study are included in the article/supplementary material. Further inquiries can be directed to the corresponding author.
